# Differential expression of hemoglobin receptor, HmbR, between carriage and invasive isolates of *Neisseria meningitidis* contributes to virulence: lessons from a clonal outbreak

**DOI:** 10.1080/21505594.2018.1460064

**Published:** 2018-05-15

**Authors:** Julien Sevestre, Seydina M. Diene, Myriam Aouiti-Trabelsi, Ala-Eddine Deghmane, Isabelle Tournier, Patrice François, François Caron, Muhamed-Kheir Taha

**Affiliations:** a Research group on microbial adaptation (EA 2656) Normandie University, UNIROUEN, Rouen, France; b Invasive bacterial Infections Unit and National reference center on meningococci, Institut Pasteur, Paris, France; c Genomic research laboratory, Service of infectious diseases, Geneva University Hospitals, Geneva, Switzerland; d Inserm U1245, UNIROUEN, Normandie University, Normandy center for genomic and personalized medicine, Rouen, France; e Infectious diseases department, Rouen University Hospital, Rouen, France

**Keywords:** Animal model, carriage, disease, genomics, iron acquisition, *Neisseria meningitidis*, virulence

## Abstract

Carriage and invasion balance in the pathogenesis of *Neisseria meningitidis* was analyzed during a recent clonal outbreak of meningococcal B in Normandy, France, that offered the opportunity to compare six isolates undistinguable by conventional typing (B:14:P1.7,16:F3-3/ST-32) isolated from invasive disease or pharyngeal asymptomatic carriage. Data from animal model (transgenic mice rendered susceptible to *N. meningitidis* infection) showed an absence of virulence for two non-capsulated carriage isolates, an intermediate virulence for two capsulated carriage isolates and a marked virulence for two capsulated invasive isolates. This differential pathogenesis well correlated with whole genome sequencing analysis that clustered both isolates of each group together, forming their own arm within the Norman cluster. Gene-by-gene analysis specified that genes involved in iron acquisition were among the elements differentially represented in cluster of invasive isolates compared to cluster of capsulated carriage isolates. The hemoglobin receptor encoding gene *hmbR* was in an ON-phase in the capsulated invasive isolates while carriage capsulated isolates were in an OFF-phase. An ON-phase variant of a capsulated carriage isolate showed enhanced virulence. These data underline the role of phase variation (ON/OFF) of HmbR in the balance between disease isolates/carriage isolates.

*Neisseria meningitidis* (Nm) is a common inhabitant of the human nasopharynx asymptomatically carried in around 3% to 25% of the population worldwide at any time. Rarely, a virulent isolate invades the bloodstream and spreads to provoke devastating life-threatening invasive meningococcal diseases (IMD) such as meningitis and meningococcemia. Phase variations create heterogenic phenotypes within a clonal bacterial population that can then facilitate switching between different virulent status [1]. Nm is also capable of spontaneous natural transformation allowing horizontal gene transfer between isolates that facilitates virulent clones to escape host immunity [1]. Thus, in most countries cases of IMD are sporadic, with a large diversity of isolates encountered during a given period, while clonal outbreaks remain uncommon.

The genesis of IMD is not completely elucidated [2]. Invasion involves host susceptibility factors and mainly bacterial virulence. The major virulence factor is the capsule which is critical for Nm to survive in the blood. Virtually all isolates from IMD express the capsule but other virulence factors are also required for full pathogenesis such as pili, lipooligosaccharide and several outer membrane proteins [3]. In addition, iron-acquisition mechanisms have a dominant role in host-pathogen interactions as iron is an essential micronutrient for Nm (involved as enzyme cofactor or regulatory element) [4]. Several genes encoding these factors undergo phase variation leading to an “ON” or “OFF” state of expression [5].

Among the 12 serogroups, defined according to the composition of the capsular polysaccharide, only 6 are responsible for almost all cases of IMD worldwide [6]. Isolates of these 6 serogroups can further be genotyped by multilocus sequence typing that analyze the DNA sequence from seven chromosomal loci and assign a sequence type (ST) for the allele combination of these 7 genes. The related STs (sharing at least 5 alleles) are then clustered into clonal complexes (CC). Few of these CCs are prevalent in IMD and are called hyperinvasive isolates [7].

Few studies used genomic approaches to address differences in virulence between different carriage and invasive isolates [8-10]. The Normandy region has recently experienced a clonal outbreak of IMD due to the spread of an hypervirulent strain of group B, serotype 14, serosubtype P1.7,16 and sequence-type ST-32 of the clonal complex 32 (B:14:P1.7,16/ST-32) [11]. During this crisis, it has been established a collection (unique in France and rare in the World) of isolates apparently identical according to conventional typing (i.e., B:14:P1.7,16/ST-32), some from IMD and others from asymptomatic oropharyngeal carriage Interestingly, some of the isolates found in asymptomatic carriers did not expressed the capsule and despite the genogroup B they were non-serogroupable (i.e., NG(B):14:P1.7,16/ST-32). Thus the aim of this study was to analyze the virulence determinants of these related isolates using whole genome sequencing (WGS) and transgenic mice rendered susceptible to Nm infection.

Six isolates from the Norman clone were compared: two capsulated isolates from invasive disease (InvB), two capsulated isolates from asymptomatic carriage (CarB) and two non serogroupeable isolates also from asymptomatic carriage, suggesting the absence of capsular polysaccharide at their surface (CarNG) (see Table 1 and supplementary material for the detail of isolates selection). By genomic typing, all the isolates showed the same fine type and MLST typing (i.e., B:14:P1.7,16:F3-3/ST-32). To gain more resolution, the six isolates were submitted to WGS and compared against two reference strains from the same clonal complex (see supplementary material for the characteristics of the two reference strains as well as the detail of genomic analysis). The phylogenic tree (Fig. 1A) based on the whole genomic sequencing and SNP (single nucleotide polymorphisms) analysis, first demonstrated that the six Norman isolates (both carriage and invasive isolates) formed a specific cluster, clearly separated from the two reference strains (with about 732 to 1528 SNPs between the six isolates). The tree also showed that both isolates of each group (invasive capsulated [Inv], carriage capsulated [CarB] and carriage non-groupeable [CarNG]) were paired together, forming their own arms within the Norman cluster (Fig. 1A). Of note non-groupeable carriage isolates diverged earlier and were located on a separated arm within the Norman cluster while the invasive and the capsulated carriage isolates appeared closely related (Fig. 1A). We next explored whether the differences between the three groups may be associated to specific genes by using a gene-by-gene analysis on the basis of either the whole pan genome (data not shown) or the 1605 genes of core genome MLST (Fig. 1B). For the two analysis similar results were obtained for both isolates of the three subgroups, as well as for a comparison with a panel of recent French invasive isolates belonging to serogroup B and CC32 (see Figure S1 in supplementary data). To focus on pathogenesis we performed a targeted gene-by-gene analysis on the basis of genes involved in meningococcal virulence such as the capsule synthesis, iron acquisition mechanisms and Maf toxin/anti-toxin systems (Fig. 1C). Interestingly, this latter comparison separated more clearly the capsulated carriage isolates from the invasive isolates (Fig. 1C). In particular, the isolates harbor hemoglobin receptor, HmbR, alone but not the HpuAB outer membrane transport system. Together, these data suggest that genetic differences among these isolates may have an impact on their respective virulence.

**Table 1.** Characteristics of the isolates used in this study.

					Genotyping				
	Year	Age (years)	Disease	Serogroup	Genogroup	PorB (serotype)	PorA-VR1	PorA-VR2	PubMLST accession (ID)
Capsulated invasive isolates (InvB)									
InvB-1483	2008	13	Purpura fulminans	B	B	14	7	16	35823
InvB-2018	2008	3	Meningococcemia with petechiae	B	B	14	7	16	35824
Capsulated carriage isolates (CarB)									
CarB-3141	2008	24	Carriage	B	B	14	7	16	35821
CarB-3644	2008	19	Carriage	B	B	14	7	16	35822
Non-capsulated carriage isolates (CarNG)									
CarNG-1126	2008	16	Carriage	NG	B	14	7	16	35819
CarNG-2963	2008	9	Carriage	NG	B	14	7	16	35820
Reference strain for NGS									
Ref-MC-58	1983		Invasive	B	B	15	7	16	240
Ref-H44-76	1976		Invasive	B	B	15	7	16	237

**Figure 1.**
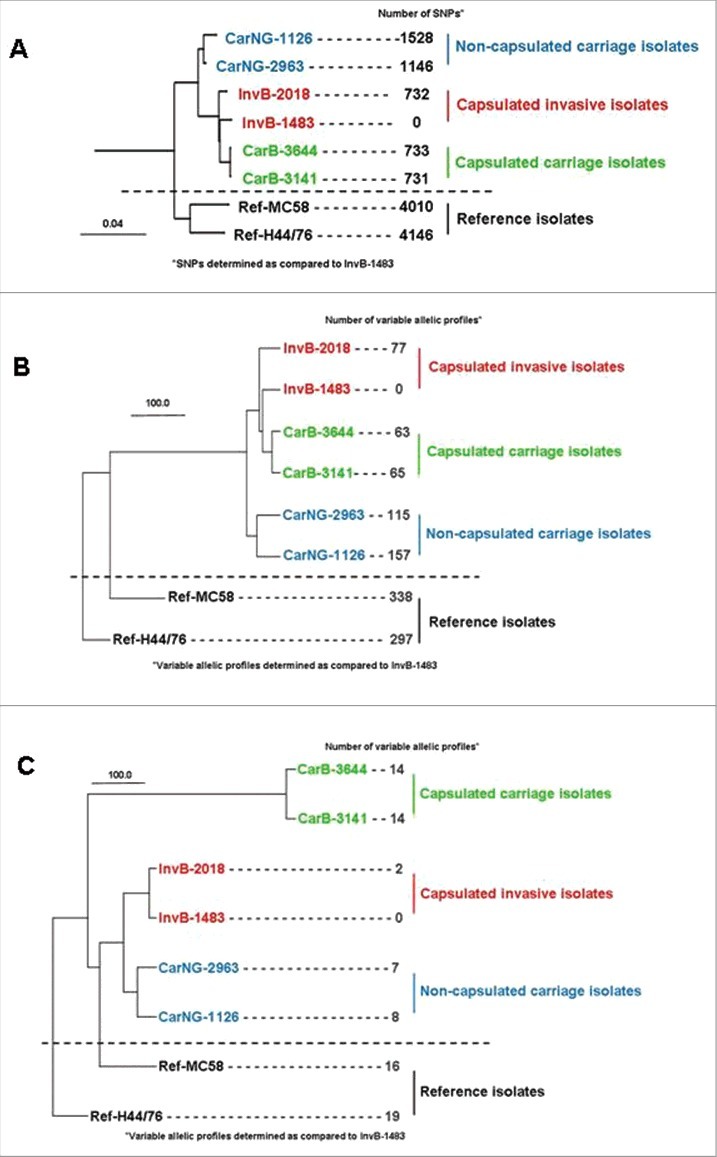
Phylogenetic trees comparing the two reference strains (MC58 and H44/76) with the six isolates from the clonal Norman outbreak: two capsulated isolates from invasive disease (InvB-1483 and InvB-2018), two capsulated isolates from asymptomatic carriage (CarB-3141 and CarB-3644) and two non-groupable (i.e., non-capsulated) isolates from asymptomatic carriage (CarNG-1126 and CarNG-2963); (A) global analysis based on whole genomic single-nucleotide polymorphisms; (B) gene-by-gene analysis based on the 1605 genes of core genome MLST; (C) gene-by-gene analysis based on 102 genes involved in meningococcal virulence (capsule genes, iron acquisition genes (*hpuAB*, *hmbR*, *fetA*, *tbpAB* and *lbpAB*) and Maf toxin/anti-toxin system) that were selected from the drop menu on the scheme box on the PUBMLST.org.

The genetic differences revealed by the genomic analysis prompted the analysis of pathogenesis in our previously described model of experimental infection in transgenic mice expressing the human transferrin (hTf) (see supplementary material for the detail of the procedure) [12]. A total of 10–18 mice were infected by a given Norman isolate and observed at 2h, 6h and 24h post-bacterial challenge. As expected, non-groupeable carriage isolates were not able to establish an infection in mice as only low numbers of bacteria were detected after 2h of infection and no detectable bacteria were identified in blood after 6h and 24h of infection. In contrast, in both animal groups infected by capsulated isolates (invasive and carriage), all mice were ill and bacteremia was detectable after 2, 6 and 24h of infection. However, invasive isolates provoked significant higher bacterial load in blood as well as higher IL-6 and KC levels than carriage capsulated isolates (details in Figure S2 in supplementary data).

WGS indicating that iron acquisition genes were among those that discriminate clusters of invasive isolates from capsulated carriage isolates, we next explored whether the differences in virulence between these two groups could be linked to ability to capture iron (see supplementary material for the detail of the analysis). Controls were first performed to demonstrate that the four isolates (InvB-1483, InvB-2018, CarB-3141, CarB-3644) grew without difference in medium supplemented with iron nitrate. It was then observed that the two groups also grew similarly in medium with human transferrin as unique iron source, and this was consistent with the fact that the four isolates harbored the same allelic profiles of the transferrin binding genes *tbpA* and *tbpB*. On the other hand, the addition of human or murine hemoglobin as unique iron source allowed the growth of invasive isolates (Fig. 2A) but not of capsulated carriage isolates (Fig. 2B). Interestingly the hemoglobin receptor encoding genes (*hmbR*) differed among the four strains in the number of G residues. The two capsulated carriage isolates showed 10 and 11 G residues in their polyguanine (G) tracts leading to an “OFF” phase of the corresponding *hmbR* genes (only multiple of G triplets are in “ON” phase). In contrast, both invasive isolates contained 15 G residues (i.e., multiples of G triplets and thus in “ON” phase) (details in Figure S3 in supplementary data).

**Figure 2.**
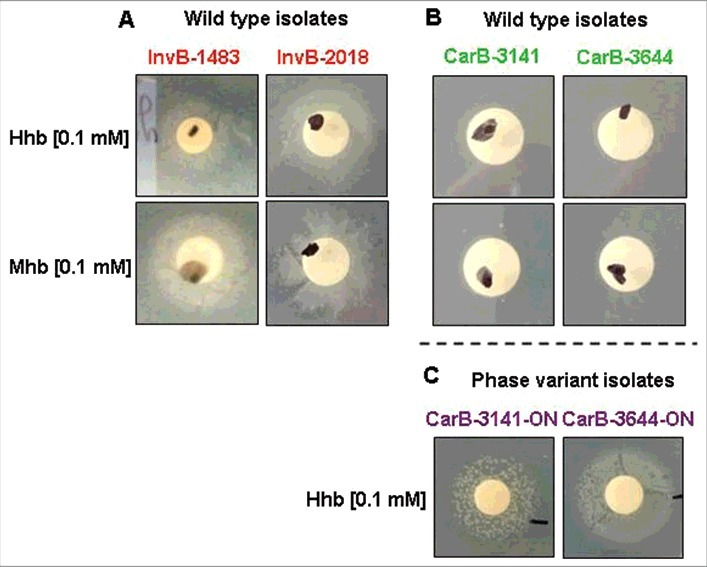
Ability or not of isolates from the clonal Norman outbreak to grow on iron depleted plate around disk impregnated with human (Hhb) or murine (Mhb) hemoglobin; (A) growth of two capsulated isolates from invasive disease (InvB-1483 and InvB-2018); (B) no growth of two capsulated isolates from asymptomatic carriage (CarB-3141 and CarB-3644); (C) growth of “ON” phase variants of the two previous carriage isolates (CarB-3141-ON and CarB-3644-ON) selected to express the hemoglobin receptor protein (HmbR).

This lead us to select an “*hmbR*-ON” phase variant of capsulated carriage isolates by plating the wild-type carriage isolates (i.e., OFF) on iron depleted plate supplemented with human hemoglobin. Bacteria from recovered colonies were controlled by Sanger sequencing for the number of G residues and found to be “ON”. These phase variants (named CarB-3141-ON and CarB-3644-ON) also recovered the ability to grow, as the invasive isolates level on iron depleted plates supplemented with hemoglobin as unique iron source (Fig. 2C).

We next tested both isogenic capsulated carriage isolates “*hmbR*-OFF” (i.e., wild-type) and “*hmbR*-ON” (i.e., phase variant) in the animal model described above (6 mice in each group). At 2h and 6h post-infection bacterial counts in the blood and levels of pro-inflammatory cytokines were significantly higher in mice infected by the phase variant isolate “*hmbR*-ON” compared to mice infected by the wild-type isolate “*hmbR*-OFF” (Fig. 3). These results suggested that the hemoglobin receptor enhanced the bacterial survival in the blood during the infection as well as the ability to provoke a higher systemic infection. We finally focused on the spleen as it is the frontline in mechanical filtration of red blood cells and in recycling hemoglobin. Compared to spleens from control mice (non-infected), infected spleens (either with OFF or ON isogenic isolates) showed more cellular density in the follicular regions of the white pulp as well as more red blood cells in the red pulp, confirming that both isolates induced a systemic infection. Of note bacteria and macrophages were only observed in the spleen of mice infected with a phase variant capsulated carriage isolate “*hmbR*-ON”, underlying their global enhanced virulence and their capacity to exceed the spleen phagocytosis ability (Fig. 4).

**Figure 3.**
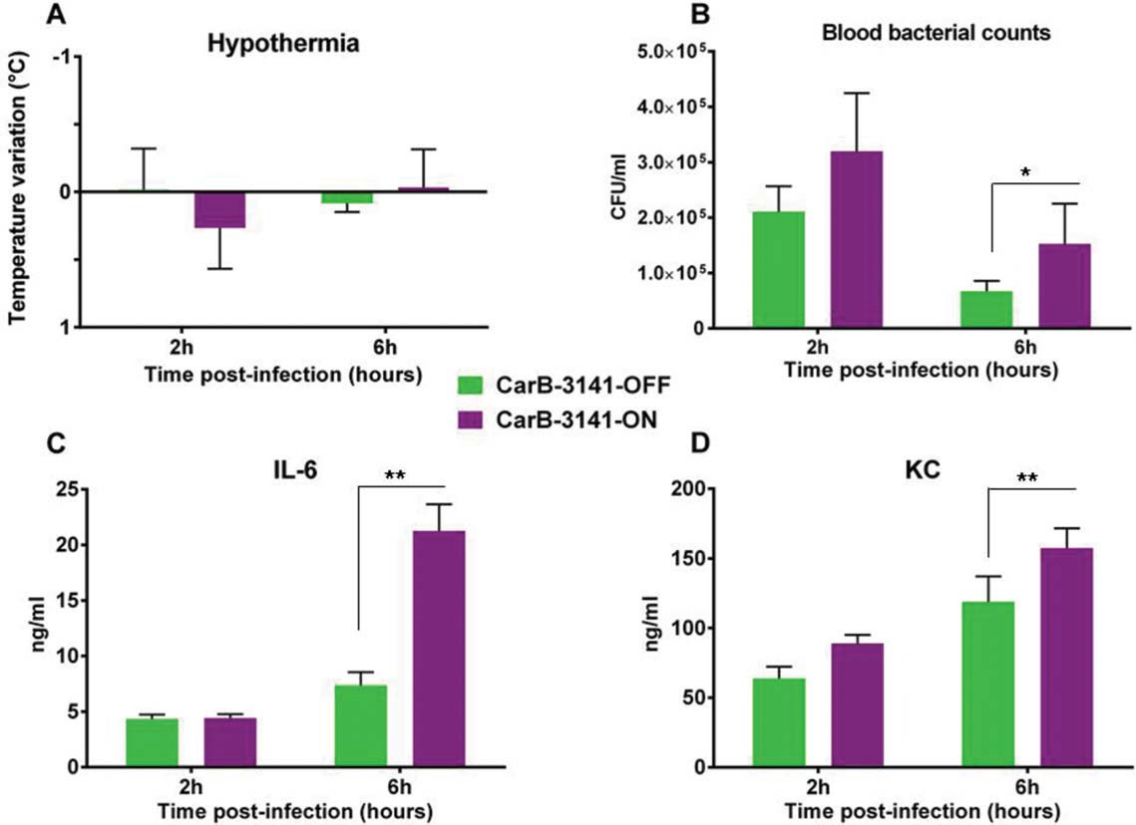
Comparative virulence in transgenic mice of wild-type capsulated isolates from asymptomatic carriage (CarB-3141 and CarB-3644) and their phase variants able to expresses the HmbR receptor protein (CarB-3141-ON and CarB-3644-ON); (A) kinetic of hypothermia post infection (i.e., symptom of infection); (B) blood bacterial counts; (C and D) levels of inflammatory cytokine IL-6 (C) and KC (D). Each point represents the mean of values achieved in 6 animals for each isolate of the given group. (*p < 0.05 and **p < 0.005).

**Figure 4.**
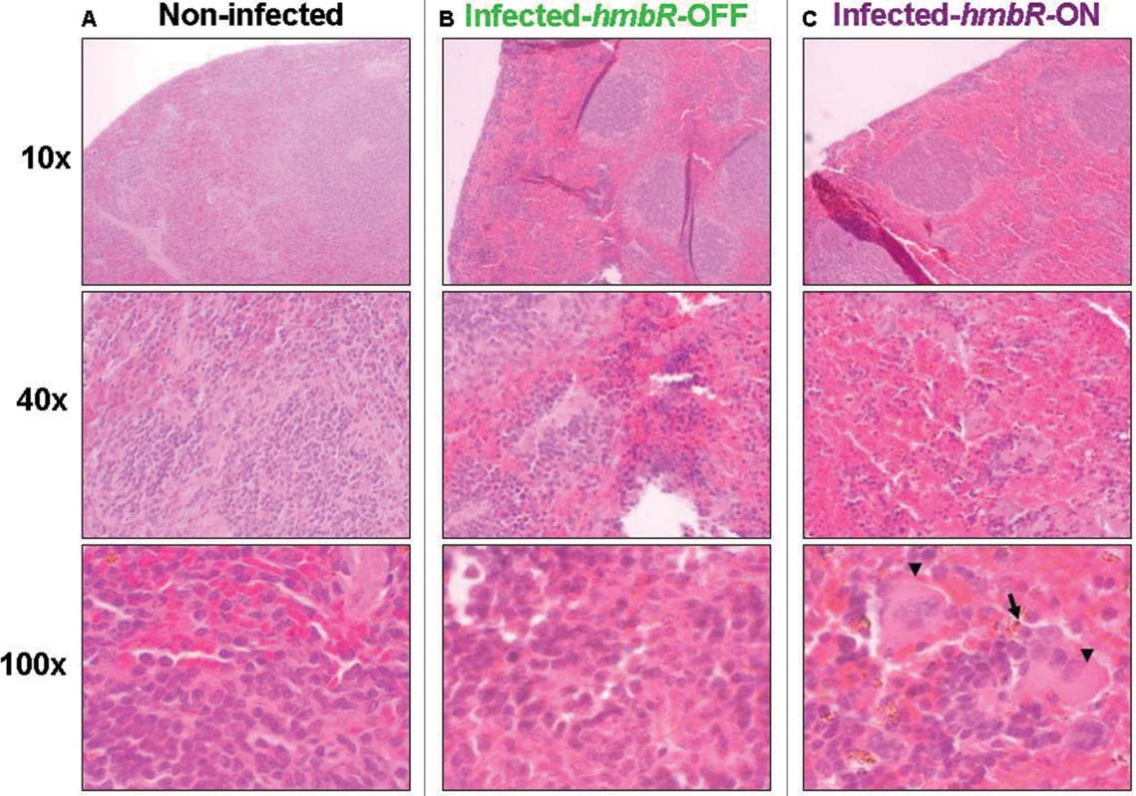
Spleen histopathology at different magnifications in transgenic mice either: (A) non-infected (negative control), (B) infected by the wild-type capsulated carriage isolate (CarB-3141-OFF) unable to express HmbR or (C) infected by the phase variant capsulated carriage isolate (CarB-3141-ON) able to express HmbR. Bacteria (arrow) and macrophages (arrow heads) are visible only for this later isolate. Presented data are typical representative pictures of three repeats for each of the two isogenic isolates (CarB-3141-OFF and CarB-3141-ON).

Thus the concordant and major results were that Nm isolates apparently identical by conventional genotyping (B:14:P1.7,16/ST-32) were distinguishable either by WGS and by their hierarchy of virulence in a transgenic mice model. In mice, non-capsulated carriage isolates failed to induce any infection, capsulated carriage isolates produced moderate disease and invasive isolates a marked systemic infection. Classically, IMD occurs upon acquisition of hyperinvasive isolates that usually show high attack rate but are rarely detected as carriage isolates. The phylogenic analysis based on virulence genes (Fig. 1C) showed that non-groupable carriage isolates remained close to the invasive isolates despite the complete loss of invasiveness in the mouse model of infection. This confirmed that the capsule expression is the major determinant of Nm virulence as its absence compromised bacterial invasiveness. In contrast, the genomic analysis on the basis of genes involved in virulence allowed more distinction among the carriage and invasive capsulated isolates that showed the same genotype (B:14:P1.7,16/ST-32) (Fig. 1C).

Our results highlight the role of iron acquisition systems in meningococci during infection. It is well known that pathogenic *Neisseria* differs from many other pathogenic bacteria by the absence of siderophores production and the direct use of human sources of iron (transferrin, lactoferrin, haptoglobin and hemoglobin) through specific receptors [4]. It is also known that changes in virulence genes may be responsible for the within-host fitness allowing Nm to be adapted for carriage and transmission [13]. Our data demonstrated a marked role of the hemoglobin receptor, HmbR, in meningococcal virulence (beside capsule expression). In Nm, the HmbR and HpuAB outer membrane transport systems allow the bacteria to use heme-loaded proteins as iron source [14,15]. Nm strains express HmbR, HpuAB or both systems [16]. Most invasive strains express HmbR alone or both heme uptake systems, as reported in isolates of the hyperinvasive clonal complex ST-11 while most of the isolates of the clonal complex ST-32 (as our isolates) only harbor HmbR [16]. Strains expressing only the HpuAB heme uptake system have been mostly described as carriage strains [16]. *hmbR* and *hpuA*, that contain a poly (G) tract, undergo phase variation [17,18]. In the current work the expression of HmbR in invasive isolates but not in carriage isolates was suggestive of involvement in enhancing virulence (ON) or association with carriage (OFF) in agreement with previous reports [19]. In particular, our data in mice showed an enhanced virulence of the “ON” variant derived by phase variation from an “OFF” carriage serogroup B isolate and the direct evidence of the HmbR implication. Previous animal experiments having focused only on blood infection by Nm had suggested that HmbR protein is not required during early stages of invasion [20]. Indeed, binding transferrin (mediated by TbpAB) seems to play an important role during blood phase of Nm infection while binding of hemoglobin could contribute to the spread of meningococci into other body compartments. The use here of a complete infected model (i.e., transgenic mice) allowed to address the role of HmbR not only in blood but also in other organs (such as the spleen). Our data clearly indicated the involvement of spleen in meningococcal disease in direct link to the expression of HmbR. The spleen is crucial to limit bacterial infection by resident phagocytes including macrophages that have also a role in facilitating iron metabolism and preventing its availability to bacteria [21]. Our histological data suggest that during infection, an important amount of blood cells is present in the red pulp where they may encounter macrophages that are also involved in removing dying red blood cells. Phase variation generates high levels of genetic diversity that enhancing bacterial fitness The expression of HmbR may allow meningococci to escape clearance by the macrophage at the sinusoidal venous system and provide iron to bacteria.

One strength of our work is to compare related meningococcal variants from a clonal outbreak in the absence of vaccination. The small number of isolates is a limitation that can be explained by the rarity of carriers of the epidemic strain in the general population. In conclusion while the virulence role of HmbR in Nm has been previously described, what is fully new in that study is to have identified changes in gene expression at a clonal level, both by WGS and in an animal model. In particular we have show directly in the model the role of phase variation (ON/OFF) of HmbR in the balance between disease isolates/carriage isolates.

## Supplementary Material

Supplementary_materials.doc
