# Supplementary material for: Differential expression of hemoglobin receptor, HmbR, between carriage and invasive isolates of Neisseria meningitidis contributes to virulence: lessons from a clonal outbreak
Source: Virulence. 2018 May 15;9(1):923–9. doi: 10.1080/21505594.2018.1460064 (PMC5955449; doi:10.1080/21505594.2018.1460064)
Supplement: Supplementary_materials.doc [file kvir-09-01-1460064-s001.doc]

**Supplementary materials:**

*Meningococcal isolates*

A total of six *N. meningitidis* isolates from the Norman B:14:P1.7,16 clone were selected in accordance with the analysis capacity by both WGS and the mouse model. Isolates were chosen to reflect the diversity of clinical settings in which they were encountered during the outbreak. Four isolates were selected from a carriage study previously performed in the first quarter 2008, at the acme of the outbreak among 3522 volunteers This previous study identified only 5 carriers of capsulated isolates sharing the complete antigenic profile of the epidemic strain (B:14:P1.7,16/ST-32) as well as 3 carriers of non-capsulated isolates lacking detectable capsular polysaccharide by agglutination (using antibodies against capsular B polysaccharide) despite their genogroup B (NG(B):14:P1.7,16/ST-32). The latter two Norman isolates provided from invasive infection expressed the complete antigenic profile of the epidemic strain (B:14:P1.7,16/ST-32). All six isolates were chosen regarding their date of detection to reflect their diversity at a given point of time. These six isolates were provided from naïve subject non-vaccinated with the MenBvac, the OMV vaccine which had latter allowed to control the outbreak . For WGS comparisons the two reference strains H44/76 (ID PubMLST 237) and MC58 (ID PubMLST 240) were chosen as they represented the closer complete genome available belonging to the same clonal complex of the Norman isolates (ST-32). H44/76 (ID PubMLST 237) was collected from an IMD in Norway in 1978 and MC58 (ID PubMLST 240) from an IMD in England in 1985. All strains were plated on chocolate agar PolyViteX (PVX agar, BioMérieux) and incubated for 18 h at 37°C in the presence of 5% CO_2_.

*Genomic analysis:*

For each Norman isolates DNA was extracted using the PurElute Bacterial Genomic Kit (Edge Biosystems) from overnight cultures, according to manufacturer’s recommendations. Genomic DNA was ethanol precipitated and resuspended in TE buffer (Tris-EDTA) for sequencing. The DNA concentration was measured using the Quant-it dsDNA High-Sensitivity Assay kit (Invitrogen). For each isolate, 3 µg of DNA were fragmented on a Covaris S220 sonicator (Covaris) yielding 200 bp fragments on average. Sequencing libraries were prepared using the SPRIworks Fragment Library Kit I (For Illumina Genome Analyzer) (Beckman Coulter) following the manufacturer’s recommendations. Genomes were sequenced on an Illumina Genome Analyzer (GAIIx) (Illumina) using 2*75bp paired-end sequencing to achieve a mean read-depth of 400x. Reads quality was assessed with FastQC v0.11.2 and these reads sequences were trimmed and filtered using the software Fastq-mcf v1.1.2. The adapters used in the library preparation were also removed. The *de novo* assembly was performed using both, Velvet assembler v1.2 and EDENA assembler v3.1. Different *k-*mer sizes were tested; 35, 45, 55 nucleotides and all others parameters set as defaults. Finals genomes were chosen regarding to different assembly results such as number of contig, the average contig size and the N50. Genome data from Illumina sequencing were uploaded to the PubMLST *Neisseria* database hosted on <http://www.pubMLST.org/neisseria> and are publically available (ID PubMLST: 35819, 35820, 35821, 35822, 35823, 35824).

WGS data were analyzed and compared against the two reference strains using a “gene-by-gene” approach available through the PubMLST Genome Comparator tool of the BIGSdb using *N. meningitidis* core genome v1.0 that includes 1605 core loci. Virulence genes were selected from the drop menu on the scheme box on the same database This approach computes the genetic distances on the basis of the different alleles among the analyzed genes. SplitsTree4 (version 4.13.1) was used to visualize the resulting distance matrices as Neighbor-net networks .

*Experimental infections in transgenic mice*

This study was carried out in strict accordance with the European Union Directive 2010/63/EU (and its revision 86/609/EEC) on the protection of animals used for scientific purposes. Our laboratory has the administrative authorization for animal experimentation (Permit Number 75-1554) and the protocol was approved by the Institut Pasteur Review Board that is part of the Regional Committee of Ethics of Animal Experiments of Paris Region (Permit Number: 75-1554). All the invasive procedures were performed under anesthesia and all possible efforts were made to minimize animal suffering.

Experimental meningococcal infections were performed in 8 week-old female congenic BALB/c transgenic mice expressing human transferrin (hTf) Mice were infected by intra-peritoneal injection of 10^7^ CFU in 0.5 ml of saline. Bacterial counts were determined in the blood sampled from the retro-orbital plexus at 2 h, 6 h, and 24 h after meningococcal challenge by plating serial dilutions of blood samples on GCB medium and were expressed in log10 CFU/ml of blood. At each point, collected sera from infected mice were tested by ELISA (R&D Systems Europe) for the titration of proinflammatory cytokines IL-6 and KC, according to manufacturer’s recommendations. Comparisons of data between different isolates were made using unpaired student’s t-test and a p-value < 0.05 was considered as significant.

In some experiments, spleens were extracted from mice after 4h-6h of infection and were fixed in 4% neutral buffered formalin for at least 24 h before processing. Tissues were embedded in paraffin by standard tissue processing methods. Sections 1 μm thick were cut and fixed on glass slides and deparaffinized. Standard hematoxylin and eosin (H&E) staining were carried out. Stained slides were examined with a light microscope (Zeiss-Axioskop) at x10, x40 and x100 magnification.

*Meningococcal growth on different iron sources*

In order to evaluate the ability of Nm to grow on various iron sources, isolates were grown on GCB agar plates supplemented with Kellogg supplements and incubated for 18 h at 37°C in the presence of 5% CO_2_. To create iron starvation, supplement 2 (containing iron nitrate) was substituted by the iron chelator, desferal, at 15, 30, 45 and 60 µM final concentration. Desferal was prepared in water at 15 mM and 30 mM. Human transferrin was prepared in water at 0.25 mM final concentration. Human and murine haemoglobin were dissolved in 100 mM NaCl at 1 mM final concentration. The haemoglobin concentration was calculated on the basis of the heme monomer. Transferrin, haemoglobin and desferal were purchased from Sigma. All solutions were filter-sterilized using 0.20 mm Millipore filters and stored at -20°C. Bacterial inoculums were calibrated and 2.10^8^ bacteria were isolated under iron depletion on plate supplemented with disk impregnate with the tested iron sources. The final concentrations tested were 5 µM for transferrin and 1 mM and 0.1 mM for haemoglobin.

(ON) isogenic variants of the *hmbR* gene in carriage (*hmbR* OFF) isolates were obtained on selective plate supplemented with hemoglobin and the number of G bases in the polyG tract within the open reading frame of *hmbR* was controlled by Sanger sequencing (Beckman coulter CEQ 8800).

**Legends of the supplementary figures**

**Figure S1.** Gene-by-gene analysis based on the 1605 genes of core genome MLST (left side) and gene-by-gene analysis based on all meningococcal genes (right side). The analysis included the six isolates of this study (IDs 35819, 35820, 35821, 35822, 35823, 35824) that were also indicated according to their category (CarB: carriage groupB, InvB: invasive isolates groupB and CarNG: carriage non groupable) and 19 invasive French isolates from the year 2013 (available in the PUBMLST.org with the following IDs: 39708, 39959, 39964, 40106, 40107, 40113, 40161, 40168, 40178, 40195, 40428, 40430, 40431, 40434, 40435, 40439, 40441, 41235, 41588),

**Figure S2.** Comparative virulence in transgenic mice of three types of isolates from the clonal Norman outbreak: capsulated isolates from invasive disease (InvB-1483 and InvB-2018), capsulated isolates from asymptomatic carriage (CarB-3141 and CarB-3644) or non-groupable (i.e., non-capsulated) isolates from asymptomatic carriage (CarNG-1126 or CarNG-2963); (A) kinetic of hypothermia post infection (i.e., symptom of infection); (B) blood bacterial counts; (C and D) levels of inflammatory cytokine IL-6 (C) and KC (D). Each point represents the mean of values achieved in 10-18 animals for each isolate of the given group. group. (* p<0.05 and ** p<0.005 ).

**Figure S3.** Sequence alignment of the region containing the homopolymeric polyG tract on *hmbR* of one reference strain (H44/76) and four isolates from a single meningococcal B clone: two capsulated isolates from invasive disease (InvB-1483 and InvB-2018) and two capsulated isolates from asymptomatic carriage (CarB-3141 or CarB-3644) The numbers of G residues in each isolate (totalized on the right) allows to deduct the phase of the *hmbR* genes: only multiple of G triplets are in ON phase.

**References**

1. Delbos V, Lemee L, Benichou J, Berthelot G, Taha MK, Caron F. Meningococcal carriage during a clonal meningococcal B outbreak in France. Eur J Clin Microbiol Infect Dis 2013; 32:1451-9.

2. Lemee L, Hong E, Etienne M, Deghmane AE, Delbos V, Terrade A, et al. Genetic diversity and levels of expression of factor H binding protein among carriage isolates of Neisseria meningitidis. PLoS One 2014; 9:e107240.

3. Caron F, du Chatelet IP, Leroy JP, Ruckly C, Blanchard M, Bohic N, et al. From tailor-made to ready-to-wear meningococcal B vaccines: longitudinal study of a clonal meningococcal B outbreak. Lancet Infect Dis 2011; 11:455-63.

4. Jolley KA, Maiden MC. BIGSdb: Scalable analysis of bacterial genome variation at the population level. BMC Bioinformatics 2010; 11:595.

5. Huson DH, Bryant D. Application of phylogenetic networks in evolutionary studies. Mol Biol Evol 2006; 23:254-67.

6. Szatanik M, Hong E, Ruckly C, Ledroit M, Giorgini D, Jopek K, et al. Experimental meningococcal sepsis in congenic transgenic mice expressing human transferrin. PLoS One 2011; 6:e22210.

7. Kellogg DS, Jr., Peacock WL, Jr., Deacon WE, Brown L, Pirkle DI. *Neisseria gonorrhoeae*. I. Virulence Genetically Linked to Clonal Variation. J Bacteriol 1963; 85:1274-9.

**Figure S1**


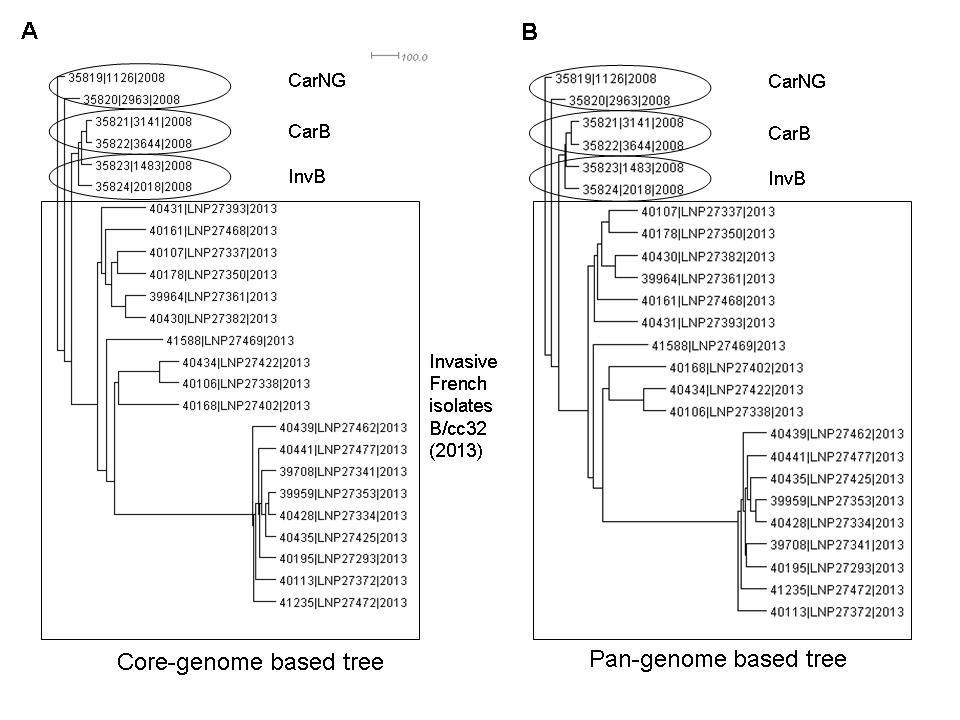


**Figure S2**

**
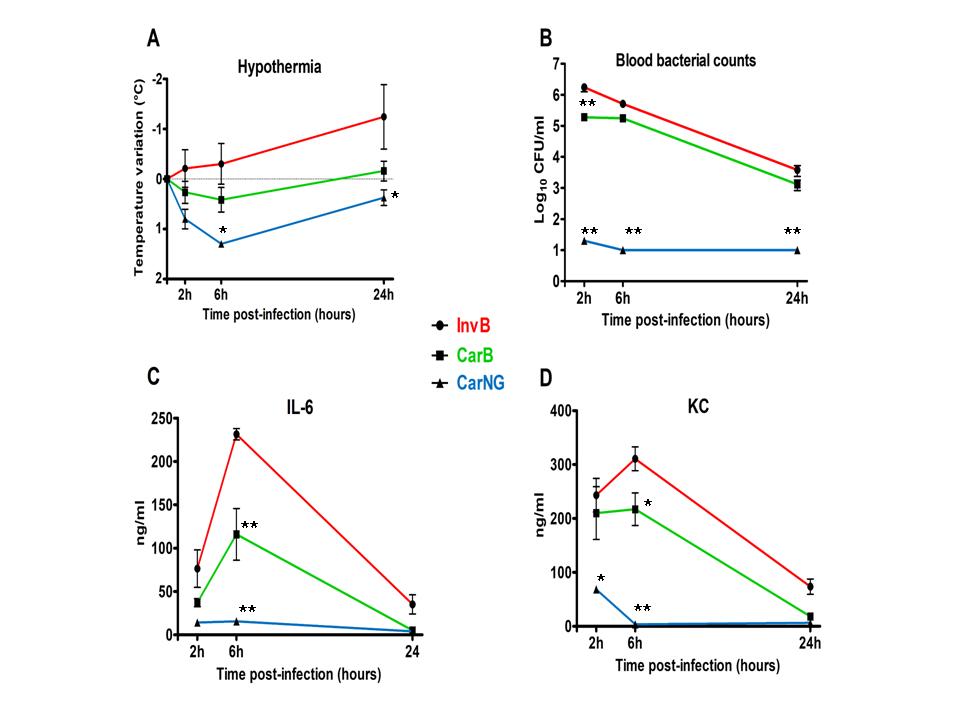
**

**Figure S3**

**
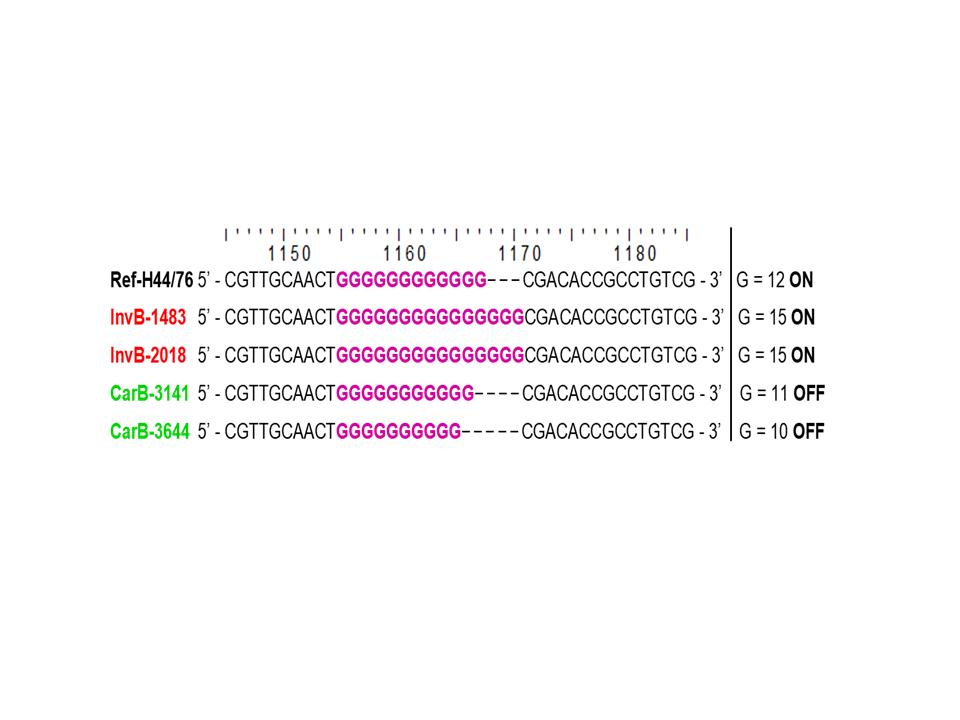
**
